# Factors Affecting Immediate Use of Contraception Among Women Hospitalised for Abortion in Two Public Hospitals in Kigali, Rwanda: A Cross Sectional Study

**DOI:** 10.24248/eahrj.v7i1.704

**Published:** 2023-07-12

**Authors:** Theodomir Sebazungu, Kenneth Ruzindana, Doee Kitessa, Urania Magriples

**Affiliations:** a University of Global Health Equity; b University of Rwanda; c University of Maryland; d University of Yale.

## Abstract

**Background:**

The 2019-20 Rwanda demographic health survey revealed an overall use of modern contraceptives of 58% but participants were not likely to use family planning in the postpartum period. Three quarters of participants intended to use contraception only after they had resumed menses and not breastfeeding. This study intended to measure post-abortion contraception uptake and to evaluate factors affecting immediate post abortion contraception uptake among patients consulting two public hospitals in Kigali, Rwanda.

**Methods:**

This is an observational cross-sectional study of women admitted for abortion in 2 hospitals' obstetric units in Kigali; the University Teaching Hospital of Kigali (CHUK) and Muhima District Hospital (MH) from November 2019 to April 2020. Admission registry was accessed daily to determine abortion admissions. After informed consent, participants underwent a standardised interview prior to their discharge from respective hospital.

**Results:**

There were 252 participants over 6 months; 88.5% were counselled for post-abortion contraception and 52% desired contraception prior to hospital discharge. Upon discharge, 70.2% of the study participants who wished immediate post abortion contraception received it before discharge and 29.8% had no contraception despite having expressed interest for immediate post abortion contraception. Being married and involving husband in choosing post-abortion contraception were significantly associated with use of post-abortion contraception.

**Conclusion:**

Post-abortion contraception uptake in 2 large public hospitals in Kigali remains low. Being married and involving husband in choosing post-abortion contraception are positive factors associated with post-abortion contraception uptake while choosing a permanent contraception is associated with not receiving any contraception at the time of discharge from hospital. There is a need to consider prescribing an alternative interim methods of contraception to women desiring permanent sterilisation.

## INTRODUCTION

Almost half of pregnancies conceived worldwide between 2015 and 2019 were unintended and 61% of those unintended pregnancies ended in abortion.^2^ During the same time frame, 38% of unintended pregnancies conceived in Africa ended in abortion.^2^ It is estimated that 58 million women of reproductive age in Africa have an unmet need for modern contraception and evidence has found 79% of all unintended pregnancy are due to unmet need for contraception.^3,4^

After an abortion, patients have a wide range of choice for contraception. Patients are allowed to use any method of family planning unless medically contraindicated. Methods that may not be safe to use immediately after giving birth may be used safely post abortion.^5^ Oral contraceptive pills, injectables, combined patch, implants, condoms, and withdrawal can be started immediately. If the subject has any infection, it is recommended that IUDs and female sterilisation is delayed until the infection is resolved/treated. If there is injury to the genital tract, female sterilisation, spermicides, IUDs, diaphragms, combined vaginal ring and cervical caps should be postponed until the injury is healed.^5^

The likelihood of initiating contraception decreases significantly when the method is not offered immediately and the risk of an unplanned pregnancy increases from 15.3% to 27.3% and subsequent abortion increases from 9.9% to 17.2%.^5,6^ Due to time needed for an additional visit for IUD insertion, even in high resource settings, only less than a third of women who plan to insert an IUD later after abortion will actually have one inserted within 6 months post abortion.^7^

In low resource settings, a number of factors have been associated with positive post abortion contraceptive uptake including; obtaining services in a private facility, seeking an induced abortion, being older than 25 years, having a first trimester abortion, choosing a medical abortion and prior use of contraception.^8^ Rates of post abortion contraception vary geographically and in Somalia, 98% of post abortion care clients were counselled for post abortion contraception and 88% accepted a contraceptive method before leaving the facility.^9^

Providing immediate access to contraception after an abortion can be challenging. A number of issues influence contraceptive use, these include; limited choice of contraceptive methods since purchasing and stocking a wide range of contraceptive methods is prohibitively expensive, especially for IUDs and implants, lack of knowledge and denial of methods to certain groups by healthcare providers, patient's lack of education about available methods, fear of side effects, partner disapproval and religious beliefs.^10,11^

The 2019-20 Rwanda demographic health survey revealed an overall use of modern contraceptives of 58% but participants were not likely to use family planning in the postpartum period.^1^ Three quarters of participants were intending to use contraception only when they had resumed menses and not breastfeeding. Furthermore, in a study on post-abortion complication in Rwanda, only 14.6% of participants planned on using contraception post-abortion.^12^ Limited data is available on the factors affecting post-abortion contraception in Rwanda.

Kigali city, which is the Capital of Rwanda accounts for one third of all induced abortions despite having only 10% of the country's reproductive age women.^13–16^

The purpose of this study was to measure post-abortion contraception uptake and to evaluate factors affecting immediate post abortion contraception uptake among patients consulting Kigali University Teaching Hospital and Muhima District Hospital.

## METHODS

### Study Design

This is a hospital based cross-sectional study that was conducted from November 2019 to April 2020 at Kigali University Teaching Hospital (CHUK) and Muhima Hospital (MH), among patients admitted for abortion.

### Study Setting

CHUK is the largest teaching and referral hospital in Rwanda. CHUK's department of Obstetrics and Gynaecology has approximately 3000 admissions and 2000 deliveries annually.^13,14^ MH has 9,000 deliveries per year and has the busiest maternity in Kigali, Rwanda.^17^

CHUK and MH have contraception services that offer counselling and provision of contraception methods. Oral contraceptive pills, injection with; depoprovera, copper IUDs, Implanon, Jadelle, male and female condoms and bilateral tubal ligation are the modern contraception available in both hospitals. Muhima hospital have Obstetricians and Gynaecologists, residents in obstetrics and gynaecology, general practitioner, intern doctor and midwives working in maternity while CHUK have obstetricians and gynaecologist, residents in obstetrics and gynaecology, and midwives

### Participants

On a daily basis, admission registries at CHUK and MH were used to determine study participants. All patients who consulted for induced or spontaneous abortion during the study period were recruited. Ectopic and molar pregnancies, pregnancies of more than 20 weeks of gestation and patients who underwent hysterectomy prior to discharge were excluded. Before participation in the study, all participants were given information about the study. All subjects gave informed written consent before participating. 11 patients who declined to participate were also excluded. They declined to participate as they had no time to be interviewed on discharge.

Data from participants was obtained through interview, conducted prior to participants' discharge from the hospital. Responses were directly recorded in the data collection form. All information obtained from the subjects was treated with confidentiality and used only for research purposes.

### Data Analysis

The analysis and interpretation of data was performed using statistical software SPSS 21 and presented as frequency tables. The chi-square (X^2^) test was used for statistical data interpretation. Statistical significance was defined as a *p* value of less than or equal to .05.

### Patient and public Involvement

10 Patients from Muhima hospital and 6 from CHUK were involved while testing the data collection form used for data collection. They were given the form and provided comments on each question asked. Furthermore, the study proposal was presented at CHUK in the department of Obstetrics and Gynaecology for review and presented again to share the study findings. Furthermore, a copy of study findings was submitted to the management of each hospital that participated in this study.

### Ethical Approval

The study was approved by University of Rwanda, School of Medicine, registration number: IRB No 417/CMHS IRB/2019 and authorised by the ethics committee of the participating hospitals.

## RESULTS

The study recruited 252 patients, 200 from MH and 52 from CHUK respectively. The age of participants ranged from 15 to 52 years with a mean age of 29.97 years. One third of the participants were unmarried. Three quarters were from Kigali city. (Table 1)

**TABLE 1:** Demographics of Study Participants

Demographic Characteristics	N (%)
Age	
<20years	23 (9.1%)
20-34years	147 (58.3%)
≥35 years	82 (32.5%)
Religion	
Protestant	149 (59.1%)
Catholic	68 (27%)
Muslim	31 (12.3%)
None	4 (1.6%)
Marital status	
Married	169 (67.1%)
Unmarried	83 (32.9%)
Residence	
Kigali city	191 (75.8%)
Rural Provinces	61 (24.2%)
Parity	
≤1	168 (67.1%)
>1	83 (32.9%)
Has at least a living child	
Yes	133 (52.8%)
No	119 (47.2%)
Had previous abortion	
Yes	35 (13.9%)
No	217 (86.1%)

88.5% of all study participants reported having been counselled for post-abortion contraception while they were in hospital and 52% desired post-abortion contraception before discharge from the hospital. 70.2% of the study participants that wished immediate post abortion contraception received it before discharge from the hospital. Implants were the most used contraception, accounting for 19.8% of the participants, followed by Depo-Provera (9.1%), IUD (4%), and oral contraceptive pills (3.6%). The rest of the participants (63.5%) did not have any form of contraception prescribed at the time of hospital discharge.

Being nulliparous or primiparous, married or cohabitating with a male partner, involving the husband in choosing post-abortion contraception, and having a spontaneous abortion of a planned pregnancy were statistically/significantly associated with use of post-abortion contraception (all *p* values <.05).

Choosing a permanent contraception was significantly associated with not receiving post-abortion contraception among the group of women who wanted to use contraception before discharge from the hospital (*p < .05*), (Table 2).

**TABLE 2:** Factors Associated with Post Abortion Contraception Uptake

	Did not Received Post abortion contraception before discharge	Received Post abortion contraception before discharge	P Value
Parity			.010
≤1	98 (58%)	71 (42%)	
>1	62 (74.7%)	21 (25.3%)	
Aborted a planned pregnancy			<.001
No	106 (77.9%)	30 (22.1%)	
Yes	54 (46.6%)	62 (53.4%)	
Married/Cohabitating with a male partner.			.021
No	61 (73.5%)	22 (26.5%)	
Yes	99 (58.6%)	70 (41.4%)	
Male partner involvement in choosing contraception			.005
No	92 (71.9%)	36 (28.1%)	
Yes	68 (58.8%)	56 (45.2%)	
Prior use of contraception in the past			.316
No	87 (66.4%)	44 (33.6%)	
Yes	73 (60.3%	48 (39.7%)	
Choosing a permanent contraception use			<.001
No	28 (23.3%)	92 (76.7%)	
Yes	11 (100%	0 (0%)	
Advanced maternal age			.053
No	101 (59.4%)	69 (40.6%)	
Yes	59 (72%)	23 (28%)	
Young maternal age			.784
No	146 (73.4%)	83 (26.6%)	
Yes	14 (60.9%)	9 (39.1%)	
Residence			.798
Urban	124 (63.9%)	70 (36.1%)	
Rural	36 (62.1%	22 (37.9%)	
Induced abortion			.001
No	106 (57.6%)	78 (42.4%)	
Yes	54 (79.4%)	14 (20.6%)	

## DISCUSSION

The study found that the overall post-abortion contraception uptake before discharge from the hospital was low at 36.5%. A report from the Rwanda Ministry of Health on expanding access to post-abortion care services in Rwanda reported a better overall post-abortion contraception uptake of 59% with variation across districts which ranged from 35% to 84%.^18^ Furthermore, studies in other African developing countries have reported post-abortion contraception uptake ranging from 61.5% to 88%.^9,19–21^

The low post-abortion contraception uptake in the 2 largest hospitals in Kigali may be due to the fact that the selected hospitals were among the busiest in Rwanda and therefore priority wasn't given to multiple sessions of counselling about post-abortion contraception. Furthermore, 29.8% of patients who wanted immediate post abortion contraception were discharged without receiving any contraception. This is concerning given that one in every 3 induced abortion in Rwanda occurs in Kigali.^16^

This study found that being married or cohabitating with a husband, involving him in choosing post-abortion contraception, and ability of women to choose contraception when the husband declined the use of family planning were the significant positive determinants of post-abortion contraception uptake. These findings align with several others studies in conducted in Africa that have shown that a woman's perception of her husband's approval of using contraception was significantly associated with contraceptive use.^22–24^ Without communicating with their partners, women who are unsure of their husband‘s opinions might decline contraception due to fear of the partner‘s opposition.^19^

Being married or cohabitating with a male partner, in addition to the husband's involvement in choosing post-abortion contraception were key factors associated with post-abortion contraception uptake. Contrary to the finding in a study done in Bahir Dar, Ethiopia where single mothers were more likely to use contraception. This study's findings are in line with several others studies in Ethiopia, Kenya and Zanzibar where married women were found to have a better post-abortion contraception uptake.^8,19–21,24^

The study demonstrated that women whose pregnancy was planned, were more likely to use post-abortion contraception. Surprisingly, there was no association with prior use of contraception, thus raising the concern whether the pregnancy was really planned. Contrary to the findings of the above cited studies in Ethiopia, Kenya and Zanzibar, plus a common believe that “prior contraception use” is a significant factor of contraception uptake, it was not significant in this study. The study rather found a negative association for women who were using contraception one month before conceiving the aborted pregnancy. We postulate that women who conceived on their preferred reversible contraception method might be reluctant to use it post-abortion and may choose a permanent contraception.

Choosing a permanent contraception was found to have a negative association with contraception uptake since all the women who opted for tubal ligation did not receive any contraception on discharge. Women who previously used any contraception and opted for a permanent contraception before discharge from the hospital who unfortunately were discharge with no contraception can partly explain the difference in findings of whether prior use of contraception is a positive factor for post-abortion contraception uptake. Not prescribing an alternative method of contraception until the tubal ligation is performed is a common finding with other studies. In Nepal 83% of women who desired tubal ligation left the hospital without contraception due to non-trained staff and lack of equipment.^25^

## CONCLUSION

To get more insight in Rwanda, future studies should analyse reasons for not receiving requested contraception, particularly, tubal ligation post-abortion and why alternatives are not discussed even if temporary. Based on this study's findings, we recommend partner involvement in post-abortion contraception to increase uptake and a follow up study to identify barriers in provision of tubal ligation post-abortion for women who need permanent contraception.

### Study Limitations

This study provides insight on factors affecting immediate post-abortion contraception uptake at discharge from CHUK and MH, however it has the following limitations. It was only performed for a 6-month period and may not reflect fluctuations that occur over time. Furthermore, it was performed at discharge from the hospital with no follow up of patients. Data of women who opted for contraception on subsequent visit or who discontinued contraception after hospital discharge were not captured. Also, the study is a cross-sectional study and was not able to confirm if patients who planned to get contraception later actually got one.
